# Exploring Interactions between the Gut Microbiota and Social Behavior through Nutrition

**DOI:** 10.3390/genes9110534

**Published:** 2018-11-06

**Authors:** Cristian Pasquaretta, Tamara Gómez-Moracho, Philipp Heeb, Mathieu Lihoreau

**Affiliations:** 1 Research Center on Animal Cognition (CRCA), Center for Integrative Biology (CBI), CNRS, University Paul Sabatier, 31062 Toulouse, France; tamara.gomez@univ-tlse3.fr (T.G.-M.); mathieu.lihoreau@univ-tlse3.fr (M.L.); 2 Laboratoire Evolution et Diversité Biologique, UMR 5174 Centre National de la Recherche Scientifique, Université Paul Sabatier, ENSFEA, 31062 Toulouse, France; philipp.heeb@univ-tlse3.fr

**Keywords:** gut microbiota, nutrition, nutritional geometry, social behavior

## Abstract

Microbes influence a wide range of host social behaviors and vice versa. So far, however, the mechanisms underpinning these complex interactions remain poorly understood. In social animals, where individuals share microbes and interact around foods, the gut microbiota may have considerable consequences on host social interactions by acting upon the nutritional behavior of individual animals. Here we illustrate how conceptual advances in nutritional ecology can help the study of these processes and allow the formulation of new empirically testable predictions. First, we review key evidence showing that gut microbes influence the nutrition of individual animals, through modifications of their nutritional state and feeding decisions. Next, we describe how these microbial influences and their social consequences can be studied by modelling populations of hosts and their gut microbiota into a single conceptual framework derived from nutritional geometry. Our approach raises new perspectives for the study of holobiont nutrition and will facilitate theoretical and experimental research on the role of the gut microbiota in the mechanisms and evolution of social behavior.

## 1. Introduction

Interactions between hosts and their microbiota, that together form the “holobiont” [1,2,3], influence various aspects of an animal’s biology, including nutrition [4] and behavior [5]. Growing evidence indicates that the microbiota can also have important consequences on the ways hosts interact with each other, for instance by triggering aggregations [6,7], guiding mate choice [8,9] or allowing kin discrimination [10]. How these complex host-microbe interactions are mediated is an open question. Recent studies point towards an effect of microbes on brain areas involved in the control of social interactions [11], or in the production of chemical signals mediating communication [12,13]. Here we argue that microbes that colonize the gut of animals may also influence a wide range of social behaviors through their impact on the nutritional needs and foraging decisions of animals.

Nutrition is central to all host-gut microbiota interactions [14]. On the one hand, gut microbes can influence the nutritional strategies of their hosts by expanding the host’s capability to digest and assimilate key nutrients [15,16], or by supplementing the host with nutrients that are difficult to find in the environment [17]. Termites, for instance, depend on complex microbial communities to exploit wood and soil nutrients that are otherwise not digestible by insects [18]. Microbes can also modify the feeding preferences of their hosts, for instance by attracting animals towards a diet that is suboptimal for the hosts but beneficial for the microbes [19]. On the other hand, nutrient intake by the hosts shapes the composition of their gut microbiota [18,20,21]. Variations in protein and carbohydrate intake modify the relative abundance of microbes in an animal’s gut [22], a change that can be observed within a few hours in mice [23], or a few days in humans [4]. 

**Figure 1:**
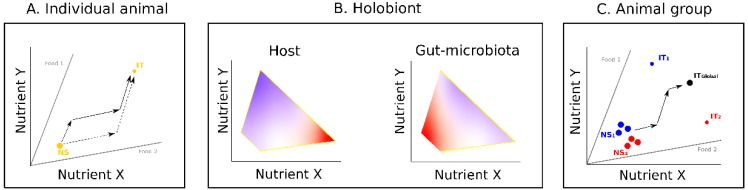
Nutritional geometry models for individual animals, holobionts, and animal groups. (**A**) Model for a hypothetical animal (modified from [25]). The nutrient space is defined by two nutrients. Nutritional rails (grey lines) represent the ratio of nutrients X and Y in two foods. Yellow dots represent the nutritional state (NS) and the intake target (IT) of the animal. In this example, foods 1 and 2 are individually imbalanced (ratio different from the animal’s IT) but collectively complementary (fall in opposite sides of the animal’s IT): The animal can reach its IT by combining its intake from the two foods. Sequences of arrows show two possible strategies. (**B**) Model for a hypothetical holobiont (modified from [26]). The performance responses of holobiont components (host and gut microbes) to nutrient intake are shown in separate heatmap landscapes. The strength of response of each holobiont component is indicated by a color code (warmer colors show strongest responses). In this example, changes in nutrient intake have different influences on the fitness of the host (maximum for high X low Y intake) and the gut microbiota (maximum for low X low Y intake) indicating the potential for a conflict in food requirements. (**C**) Model for a hypothetical group of animals (modified from [28]). The group is composed of two sub-groups of highly interacting individuals (blue and red circles). In this example, each individual has its own NS but individuals of the same sub-group have the same IT (sub-group 1: IT_1_; sub-group 2: IT_2_). All individuals are socially attracted and feed together on the same foods. Animals must trade-off between choosing food to address their own nutritional needs and following others. This can be achieved by collectively reaching a group-level intake target (IT global) midway between the two sub-group ITs.

Over recent years, state space models of nutritional geometry [24,25] (Figure 1) have been increasingly used to study the nutritional interactions between gut microbes and their hosts, an approach known as “nutritional immunity” [14]. In these models, animals and foods are represented in a nutrient space defined by two or more key food components for the animal (typically, but not exclusively, the macronutrients carbohydrates, proteins and fats; for a recent review see [26]). Foods are radials defined by the ratio of the food components under consideration (nutritional rails). The animal is characterized by a nutritional state (NS), a snapshot of its nutritional condition, and by an intake target (IT), an optimal state at which fitness is maximized. The challenge for the animal is to consume available foods in amounts and balances allowing it to reach and maintain its IT [25]. Knowing the nutritional composition of foods, and the NS and the IT of the animal, it is thus possible to predict the feeding strategy that will most efficiently enable the animal to meet its nutritional needs (see examples in Figure 1A).

Nutritional immunology studies have extended this framework by considering microbes as trophic analogues of animals [27]. In this approach, an animal’s gut microbiota can be modelled in a nutrient space and characterized by a specific NS and IT [26]. Here, however, the NS of the gut microbiota varies with the nutritional behavior of its host. Using nutritional geometry designs, the performance responses of the host and the gut microbiota can be derived by experimentally mapping the expression of fitness traits of both holobiont components in separate nutrient spaces (see examples in Figure 1B). Therefore, in principle, it is possible to predict how the nutrient intake of a host will affect its fitness and the development of different microbe types (strains or species) in its gut [26].

Concepts of nutritional geometry have also been used to explore the effects of nutrition on social interactions, an approach called “social nutrition” [28,29,30]. Here, nutrition models are used to study how nutrition affects many social behaviors and structures, such as collective movements [31], cooperative foraging [32], or reproductive division of labor [33]. In social species, individuals must often trade-off between choosing foods that address their own nutritional needs and following others’ choices to maintain social cohesion, which can generate a variety of social responses [28]. These interacting effects between nutrition and social behaviors can be modelled by considering all individuals constituting a social group in a common nutrient space (Reference [28]; Figure 1C). Each individual is defined by its own NS and attempts to reach its IT. The ability of the different group members to do so depends on the nature and the frequency of their social interactions (e.g., attraction [34], competition [35,36], cooperation [37]). In social insects, for example, nutrient balance is achieved collectively by the foragers that collect nutrients to reach a colony IT composed of the different ITs of all colony members, including the other workers that need carbohydrates for energy, and the larvae that need proteins for growth [38].

In this review, we argue that concepts of nutritional geometry can bring new fundamental insights into emerging research on microbiota and social behaviors, by integrating nutritional interactions at different levels (between hosts and their gut microbes, and among hosts) in a common theoretical framework. We hypothesize that, by acting on the nutritional decisions of their hosts, gut microbes can influence a wide range of social behaviors, which in turn impact the composition of the gut microbiota communities. We develop this hypothesis, by first reviewing research demonstrating the links between the gut microbiota and the feeding behaviors of their hosts. We then describe how these multi-level interactions can be modelled in nutritional geometry to generate new empirical predictions about the influence of the gut microbiota on social behaviour. External microbes living on the surface of the animals may also influence a range of social behaviors [10,39,40,41,42,43]. However, it is unlikely that nutritional requirements of these microbes influence host nutritional decisions; at least before they are ingested by individuals, for instance through self-cleaning behaviors [44]. For these reasons, here we only focus on microbes that colonize the gut.

## 2. Influence of Gut Microbiota on Host Nutrition

The gut microbiota can modify the feeding preferences of their host. For instance, gnotobiotic fruit flies experimentally colonized with a specific bacterium species, and given a choice between several food sources with the same nutritional composition but seeded with different bacteria species, show an attraction towards the source seeded with the bacterium species present in their gut, whereas axenic (microbe free) flies show no preference [19]. This gut bacteria-mediated food preference is expressed by flies even if the food source is suboptimal (nutritionally imbalanced) and unseeded by bacteria, suggesting that the microbiota can cause important fitness costs by pushing the host away from its intake target [19].

How exactly gut microbes affect host nutritional behaviors is not clear. A major hypothesis is that microbes act through the gut-brain axis, a communication channel between the gut and the central nervous system [45,46], which involves regulatory processes that modify the gut microbial compositions and brain activity [46,47,48]. The gut-brain axis is involved in stress regulation, emotion, social bonding (reviewed by [49]), and eating disorders [50,51,52]. In mice, gut bacteria maintain the expression of receptors of fat that are altered in axenic individuals [53]. Axenic mice also show an increase in the number of sweet taste receptors in the gastro-intestinal tract compared to wild type non-axenic mice [54].

**Figure 2:**
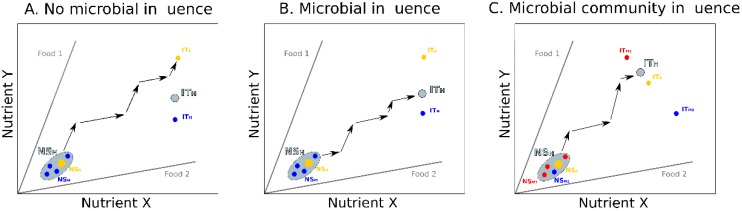
Influence of the gut microbiota on host nutrition. In these hypothetical examples, hosts and microbes are represented in a common nutrient space defined by two nutrients. Food types (grey lines) are defined by their ratio in nutrients X and Y. The animal host (yellow dot) and the microbes (blue and red dots), together forming the holobiont (grey surface), are charatcerized by their own nutritional states (animal: NS_A_; microbe: NS_M_; holobiont: NS_H_) and intake targets (animal: IT_A_; microbe: IT_M_; holobiont: IT_H_). (**A**) The animal is colonized by a single microbe type that does not influence its feeding behavior. The animal can reach the IT_A_ by combining its intake of the two complementary foods (black arrows). The optimal IT_H_ for both holobiont components is never reached. (**B**) The animal is colonized by a single microbe type that influences its feeding behaviors. The animal can reach an IT_H_ that falls between IT_A_ and IT_M_ depending on the relative influence of the host and the microbes on feeding decisions. (**C**) The animal is colonized by two microbe types (assemblage of microbes) that both influence its feeding behavior. The animal can reach an IT_H_ that falls between IT_A_, IT_M1_ and IT_M2_ depending on the relative influence of the host and the two microbe types on feeding decisions. In this example, microbe type 1 has a stronger influence on the feeding decisions of the host than microbe type 2. The same logic can be extended to richer microbial communities where a global effect of the microbial community can affect the animal feeding behavior.

In principle, these complex interactions between gut microbiota composition and host feeding behaviors can be investigated using nutritional geometry. Previous studies have explored these effects by considering hosts and microbes in separate nutrient spaces (e.g., [26]; Figure 1B). Here we propose to integrate both components of the holobiont in a single nutrient space (Figure 2). In this approach, the host and the gut microbes have their own NS (animal host: NS_A_, microbes: NS_M_) defining a global holobiont nutritional state (NS_H_). The two components also have their own IT (animal host: IT_A;_ microbes: IT_M_), defining a holobiont intake target (IT_H_). Every time the host ingests food, its microbial community is affected and can potentially change in composition (e.g., relative abundance of microbes or types of microbes [22]), which dynamically influences both the NS_H_ and the IT_H_.

Several theoretical scenarios can then be considered. If the microbiota has no effect on the host feeding preferences, the host selects foods in order to reach and maintain its IT_A_ (Figure 2A). In this case, the IT_H_ cannot be reached. If the microbiota affects the host feeding preferences (e.g., [19]), the feeding behavior of the host mirrors the compromise between selective forces imposed by the host and the microbiota to reach their own intake targets. Nutrient intake by the host then depend on the relative influences of the two holobiont components in determining feeding decisions. If the nutritional interests of the host and its microbiota are in conflict any approach of the IT_H_ towards the IT_A_ moves the IT_M_ away from its optimum, and vice versa. Depending on the composition of the microbial community, some microbe types may have more influence than others on the host behavior, and drag the nutrient intake towards their own IT_M_. Consequently, if only one microbe type colonizes the host (e.g., controlled experiments using gnotobiotic animals), host nutrient intake should follow nutritional rails leading somewhere between the IT_A_ and the IT_M_ and eventually ending on IT_H_ (Figure 2B). In more natural conditions, where several microbe types colonize the host and compete (or cooperate) among themselves [55], the host nutrient intake should fall between the IT_A_ and the different IT_Ms_ of all the microbe types, weighted for their relative influence on host feeding decisions (Figure 2C). In all cases, the microbiota can influence the NS_A_, by expanding the host’s capacity to assimilate nutrients [15,16], or by supplementing the host with new nutrients [17]. This may modify the host feeding choices, and lead the holobiont to reach the IT_H_. These interactions are expected to be highly dynamic since the composition of the gut microbiota can rapidly change as the host consumes different foods [4,22].

## 3. Integrating Gut Microbes, Host Nutrition and Social Behavior

In addition to influencing individual host behavior, microbes can affect the type and frequency of social interactions among hosts, for instance by triggering aggregations (e.g., drosophila [6]; cockroaches [7]), guiding sexual interactions (e.g., hyenas [9], drosophila [8]), or mediating kin recognition (e.g., ants [39]). Conversely, social behaviors can facilitate microbe transmission among hosts and shape their microbial communities by increasing the diversity of microbe types per individual [56] and by homogenizing communities across individuals [13]. Hosts that live together or interact frequently tend to have more similar microbiota than those that rarely encounter (e.g., baboons [57], meerkats [58], birds [59,60], bees [61]).

Based on these observations, we argue that gut microbes can play a major role in mediating host social interactions. By acting on the nutritional decisions of animals, microbes may affect the nature and frequency of social interactions among their hosts within groups or populations. Transfer of microbes between social partners may homogenize gut microbiota profiles within groups and further promote these interactions. Below we build on concepts of nutritional geometry to integrate host-microbiota interactions ([14,26]; Figure 1A,B and Figure 2) and host social behavior ([28,29]; Figure 1C) in a common framework.

**Figure 3:**
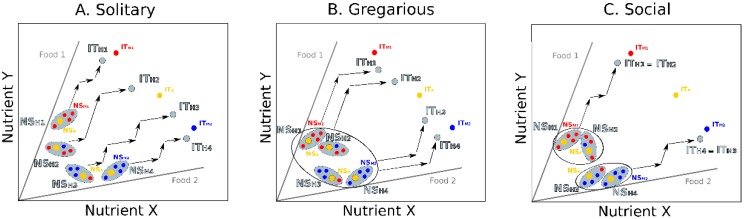
Integrating microbiota, host nutrition and social behavior. Populations of holobionts are represented in a nutrient space defined by two nutrients. Foods (grey lines) are defined by their ratio in nutrients X and Y. Animal hosts (yellow dots), gut microbes (blue and red dots), and holobionts (grey surfaces) are characterized by their nutritional states (animal: NS_A_; microbe: NS_M_; holobiont: NS_H_) and intake targets (animal: IT_A_; microbe: IT_M_; holobiont: IT_H_). Black arrows illustrate the feeding decisions of each holobiont or sub-groups of interacting holobionts. Three hypothetical scenarios depict the expected nutritional interactions between gut microbes and hosts in groups of increasing social complexity. (**A**) In solitary species, where individuals feed independently and do not interact, the diversity of environmental microbes may generate a high variability in the NS_H_ and the IT_H_ of the different holobionts. Each holobiont can reach its IT_H_ by alternating its intake of the two complementary foods. (**B**) In gregarious species, where individuals feed together and regularly interact, horizontal transmission of microbes may reduce inter-holobionts variability of NS_Hs_ and IT_Hs_. In this example where individuals do not interact evenly, horizontal transmission favors the emergence of sub-groups (black circles) of highly interacting individuals with similar gut microbiota that can collectively reach their IT_H_. (**C**) In advanced societies, where individuals with different nutritional needs (e.g., young and adults, queens and workers) permanently co-occur, differential social interactions and continuous homogenization of gut microbiota through social contacts favor the emergence of classes of highly interacting individuals with distinct NS_Hs_ and IT_Hs_ (e.g., castes). Holobionts can cooperate with other holobionts from the same class to reach their common IT_H_.

In this approach, multiple hosts and microbes are modelled in a single nutrient space, together forming a population of holobionts (Figure 3). As previously, holobionts have their own NS_H_ and IT_H_. Each time an animal ingests food, its microbial community can be altered, thereby affecting both the NS_H_ and the IT_H_. The microbiota composition can affect the nutritional decisions of each animal host depending on whether or not microbe types influence their feeding preferences. Here, however, the feeding decisions and microbiota composition of each holobiont also depend on the social interactions between holobionts. Social attraction, for instance, is expected to favor collective feeding and physical contacts leading to the homogenization of NS_H_ and IT_H_ among holobionts. In contrast, competition or social avoidance are expected to generate a high variability in NS_H_ and IT_H_ among holobionts, due to a reduced transmission of microbes.

Using this conceptual representation helps identify theoretical scenarios about how the gut microbiota may affect social interactions in groups exhibiting various levels of social complexity. In solitary species, where individuals rarely interact, the microbiota can be vertically transmitted across generations from mothers to offspring [62,63] or acquired from the environment [64]. In this case, we expect the NS_H_ and the IT_H_ of independently feeding individuals to be firstly influenced by vertical transmission and then primarily dependent on food intake, resulting in high variability across the nutrient space (Figure 3A). The degree of variability of the NS_H_ and the IT_H_ across the population of holobionts should mirror the diversity of the microbes available in the environment and the breadth of the hosts’ diet. This variability may be increased if microbes influence the nutritional decisions of the animal hosts, for instance by modifying their feeding preferences towards different food types [19]. In highly heterogeneous environments, such microbial influence on host feeding behaviors may further reduce the frequency of social interactions among animals searching for different food types, and reinforce the tendency for solitary behaviors.

In gregarious species, where individuals interact more frequently and often eat in groups, both vertical transmission and horizontal transmission (through social contacts) of microbes may occur, thereby reducing the variability in microbiota composition among hosts of the same group [65]. In this case, we expect a low variability of the NS_H_ and the IT_H_ across the holobionts (Figure 3B). If hosts do not interact evenly among each other, for instance following kinship or familiarity biases [66,67,68], the homogenization of the microbiota may give rise to sub-groups of interacting holobionts with more similar NS_Hs_ and IT_Hs_, a mechanism further promoting social interactions within these sub-groups [7], and the emergence of a social structure.

In the most complex societies, characterized by strong social cohesion, frequent interactions and durable social structures, both vertical and horizontal transmissions of microbes occur. In such groups, microbes often need repeated inoculations to reach a mature functional form or assemblage useful to the host [69]. Here, continuous homogenization of gut microbiota through social contacts and differential social interactions among social partners may favor the emergence of specialized classes of highly interacting individuals with distinct NS_Hs_ and IT_Hs_. This may be the case in social insects, where individuals at different developmental stages (e.g., larvae and adults) and belonging to different castes (breeders and workers) are characterized by highly divergent NS_Hs_ and IT_Hs_ [32,70,71]. Intra-class social interactions can lead hosts belonging to the same class towards situations where similar feeding choices strongly affect the microbiota composition, which in turn affect the hosts social behaviors. This feedback creates class specific NS_Hs_ from which the different holobionts cannot “escape”, since the specific condition of the hosts leads to an increase in the IT_H_ distances (Figure 3C). At this stage, holobionts head on a nutritional rail that can be costly to change thus impeding movements of individuals from one class to another. A typical example is observed in ant colonies where individuals from different castes have different nutritional needs and transitions from one caste to another is accompanied by morphological, physiological and behavioral changes [72]. Alternately, intra-class variations of host nutritional choices can incur costs to the resident microbiota that can be detected by the loss of certain bacterial types, or the replacement by others. It can thus be predicted that associations between hosts and their gut microbiota are more specialized and stable in social hosts than in solitary hosts.

## 4. Conclusions

**Figure 4:**
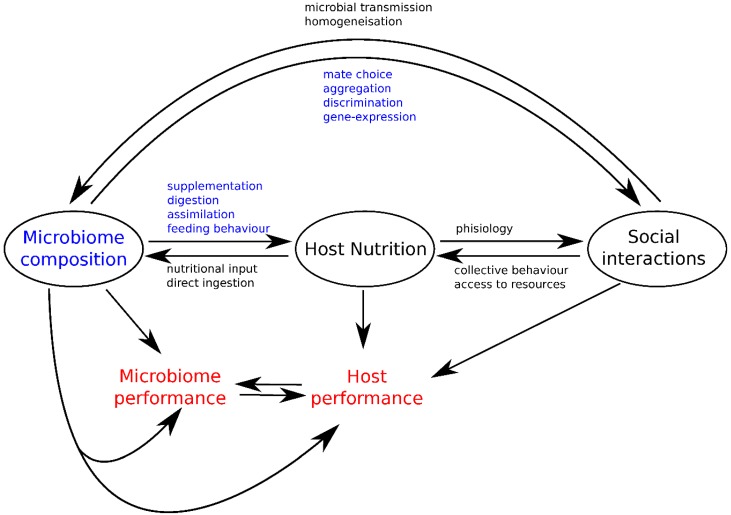
Nutritional interactions between the gut microbiota, host feeding strategies and host social interactions.

Nutritional geometry is a powerful approach in the study of nutritional interactions across organizational levels from molecules to individuals, groups and communities [28,73,74]. As we have described above, concepts of nutritional geometry can help in modelling how gut microbiota composition, host feeding behaviors and host social interactions integrate within a single theoretical framework. By identifying nutritional interactions and feedback loops between these three different components, this approach allows the formulation of new hypotheses to explore the role of the gut microbiota in the mechanisms and evolution of host social behaviors (Figure 4).

While nutritional studies have typically considered animal food intake and performance responses as a proxy of the intake target of an individual, growing evidence shows that these preferences vary among individuals, presumably because animal feeding decisions are influenced by several additional factors, including (but not only) its microbiota and social interactions [25]. Host nutritional choices can modify the relative abundance of microbe types in their gut by varying nutritional input available for microbial development [22] or through direct ingestion [18]. In turn, the gut microbiota composition can affect host nutrition by expanding [15], digesting and assimilating key nutrients for the host [17], or by dragging the host towards suboptimal diets [19]. Microbiota can intervene in gene-expression involved in social propensity [75], guiding mating choices [8,9], promoting aggregations [6,7], and mediating kin discrimination [39]. Conversely, social interactions favor the homogenization of the gut microbiota by increasing microbial transmission across individuals in the population [13,56]. Social interactions can also indirectly affect microbiota composition via the impact of collective behaviors during foraging [76] or differential access to resources [77].

The interplay between gut microbiota, host nutrition, and social interactions (Figure 4) emphasizes the need to study host-microbiota interactions using a more integrative approach, by considering populations of hosts and their feeding decisions. Using nutritional geometry, we derived speculative scenarios about how gut microbes may affect host social interactions and how host social interactions might constrain feeding decisions, and as a result, microbiota composition. The challenge for future studies will be to carry out experiments on specific components in order to examine the mechanisms underlying co-evolutionary feedback acting between hosts and their microbiota [55]. Deconstructing the nutritional needs and performance responses of the different components of host-microbe associations will help tackle important ongoing discussions about the relevance of the concept of the holobiont, whilst looking for potential evolutionary conflicts between microbes, host nutrition and social behaviors.

Computational models of nutritional geometry have already begun to explore nutritional interactions in populations of agents [73,78] and integrate multiple levels of interactions (e.g., host-pathogen interactions [37]). From an experimental point of view, it is possible to design manipulative experiments on host and microbiota nutrition, using behavioral assays or physiological measures on organisms provided with artificial diets [19]. Although it remains difficult to measure the intake target of microbe types in their natural host environment (where several microbes interact within assemblages), manipulating the microbiota composition under controlled conditions, using axenic [79,80] and gnotobiotic [19] animals, can help estimate the intake target of the animal host and how it is influenced by the microbiota. In the field, it is possible to reconstruct the gut microbiota composition of an animal and its nutrient intake by monitoring the animals’ food choices, and analyzing food samples and feces composition (see [81,82] for details). At the collective level, social network analyses constitute powerful tools to assess how gut microbiota influence social interactions and structures [83,84]. By quantifying social interactions and characterizing the nutritional needs of experimentally manipulated individuals, it is possible to picture the associations between microbiota-induced phenotypic changes (e.g., variation in holobiont nutritional needs) and the temporal dynamics of social interactions. This experimental approach might also be applied in species reared in their natural environments. For instance, using animal models that naturally show flexibility in their social behavior, either at different developmental stages or in different environments (i.e., social tipping points; see Reference [85] for details), it is possible to evaluate the variation of the onset of changes induced by the microbiota. Additionally, experimental manipulation of host nutrition and control of their social interactions, for instance using model organisms [19,86,87], can also help clarify the role of diet and social behavior in defining the effects of gut microbiota in hosts [44,60,88]. Since the basic principles of nutrition are shared by virtually all animal species [25], results from this approach may bring insights into the influence of the microbiota in the mechanisms and evolution of social life across a wide range of organisms.

Microbiota composition can be a key component shaping animal behaviors [5]. Thus, the interplay between microbiota and the host is central to understand multiple host interactions. The behavioral plasticity observed in the host can be seen as a combination of the interactions between environmental factors, and the genomic repertoire of the microbiota (i.e., microbiome) and the one of its host [89]. It is thus the holobiont itself that is plastic and able to cope with environmental and biological stressors (e.g., parasite infections [61,90]), possibly driving selection of both hosts and microbiota communities. Nutritional choice is one possible evolutionary channel of host diversity [91], shaping gut microbiota specialization [92], and driving the emergence of diverse social organizations across multiple taxa [65]. We hope that quantitative analyses of these interactions, as facilitated with nutritional geometry, will encourage future research in this direction.
